# Endothelial CaMKII as a regulator of eNOS activity and NO-mediated vasoreactivity

**DOI:** 10.1371/journal.pone.0186311

**Published:** 2017-10-23

**Authors:** Shubha Murthy, Olha M. Koval, Juan M. Ramiro Diaz, Santosh Kumar, Daniel Nuno, Jason A. Scott, Chantal Allamargot, Linda J. Zhu, Kim Broadhurst, Velarchana Santhana, William J. Kutschke, Kaikobad Irani, Kathryn G. Lamping, Isabella M. Grumbach

**Affiliations:** 1 Department of Internal Medicine, Carver College of Medicine, University of Iowa, Iowa City, Iowa, United States of America; 2 Iowa City Veterans Affairs Healthcare System, Iowa City, Iowa, United States of America; 3 Central Microscopy Research Facility, Office of Vice President of Research and Economic Development, University of Iowa, Iowa City, Iowa, United States of America; 4 Department of Pharmacology, Carver College of Medicine, University of Iowa, Iowa City, Iowa, United States of America; University of Southampton, UNITED KINGDOM

## Abstract

The multifunctional Ca^2+^/calmodulin-dependent protein kinase II (CaMKII) is a serine/threonine kinase important in transducing intracellular Ca^2+^ signals. While *in vitro* data regarding the role of CaMKII in the regulation of endothelial nitric oxide synthase (eNOS) are contradictory, its role in endothelial function *in vivo* remains unknown. Using two novel transgenic models to express CaMKII inhibitor peptides selectively in endothelium, we examined the effect of CaMKII on eNOS activation, NO production, vasomotor tone and blood pressure. Under baseline conditions, CaMKII activation was low in the aortic wall. Consistently, systolic and diastolic blood pressure, heart rate and plasma NO levels were unaltered by endothelial CaMKII inhibition. Moreover, endothelial CaMKII inhibition had no significant effect on NO-dependent vasodilation. These results were confirmed in studies of aortic rings transduced with adenovirus expressing a CaMKII inhibitor peptide. In cultured endothelial cells, bradykinin treatment produced the anticipated rapid influx of Ca^2+^ and transient CaMKII and eNOS activation, whereas CaMKII inhibition blocked eNOS phosphorylation on Ser-1179 and dephosphorylation at Thr-497. Ca^2+^/CaM binding to eNOS and resultant NO production *in vitro* were decreased under CaMKII inhibition. Our results demonstrate that CaMKII plays an important role in transient bradykinin-driven eNOS activation *in vitro*, but does not regulate NO production, vasorelaxation or blood pressure *in vivo* under baseline conditions.

## Introduction

The multifunctional calcium/calmodulin-dependent kinase II (CaMKII) is a ubiquitously expressed serine/threonine kinase that decodes intracellular Ca^2+^ oscillations into signaling events [1]. Thus, the role of CaMKII in excitable tissues such as neurons and cardiac myocytes in health and disease has been extensively studied [2, 3]. CaMKII has emerged as a regulator of ion channels, ER and mitochondrial Ca^2+^ uptake and Ca^2+^-dependent gene transcription [2, 4]. Numerous studies support the concept that the short-term activation of CaMKII promotes intracellular Ca^2+^ homeostasis, while its chronic activation in different cardiovascular diseases facilitates disease progression [5].

Recently, a role for CaMKII in vascular diseases such as hypertension and remodeling after injury has emerged [6–10]. Whereas the findings are mostly based on data in vascular smooth muscle cells, the role of CaMKII in endothelial function is currently poorly understood. Numerous obstacles have hampered our progress in dissecting CaMKII function in endothelium. First, several isoforms and splice variants of CaMKII are known. While one study identified CaMKII isoform δ6 as the predominant isoform in endothelial cells [11], another group surprisingly reported heterodimers of CaMKII isoforms α and β [12], which were previously believed to be restricted to neuronal tissues [13]. Considering these unresolved discrepancies, it is difficult to conclusively interpret data obtained with isoform-specific knockdown. Second, many published experiments aimed at characterizing the effects of CaMKII inhibition on endothelial function have been conducted with the compound KN-93 [14, 15] that has well-characterized CaMKII-independent effects on ion channels, such as L-type Ca^2+^ and voltage-dependent K^+^ channels [16]. KN-93 also blocks CaMKI and CaMKIV [16]. Third, though some canonical CaMKII substrates have been characterized in contractile tissues [17], targets such as phospholamban are not strongly expressed in endothelium, and a systemic approach to identify CaMKII substrates selectively in the endothelium has not been conducted. While some evidence suggests the endothelial nitric oxide synthase (eNOS) is a phosphorylation target of CaMKII [15, 18], a later study did not substantiate that CaMKII regulates eNOS activity [19]. This, together with the fact that CaMKII activity in these studies was examined using KN-93 calls for further clarification.

These and further *in vitro* studies proposing a role of CaMKII in stress fiber formation and endothelial permeability were conducted in endothelial cells or endothelium-derived cell lines [11, 20] that undergo phenotypic changes once in culture. Thus, these data cannot be extrapolated to CaMKII function *in vivo* without further experimental validation. However, models to study CaMKII function specifically in endothelium are currently missing, and available data are limited to two studies of vasoreactivity with the pharmacologic inhibitor KN-93 [15, 21].

The current study sought to overcome some of these obstacles by developing novel transgenic models of CaMKII inhibition in endothelium. Here, we used the approach of overexpressing CaMKII inhibitor peptides AC3-I [22] or CaMKIIN [23] selectively in endothelium *in vivo*. The inhibitor peptides serve as pseudo-substrates and inhibit all CaMKII isoforms or splice variants. Their potency and specificity to inhibit CaMKII have been well characterized [24]. Moreover, transgenic models that express AC3-I or CaMKIIN in cardiac myocytes, respiratory epithelium and smooth muscle have been used successfully by our group and others to delineate CaMKII function in heart, lung and vascular disease [5, 25, 26]. Using our novel endothelium-selective transgenic CaMKII inhibitor models, we defined the role of CaMKII in baseline blood pressure, NO production and vasodilation in this study. Furthermore, we performed mechanistic studies in cultured endothelial cells with CaMKII inhibition.

## Materials and methods

### Materials

Bradykinin, acetylcholine, L-N^G^-Nitroarginine methyl ester (L-NAME), L-N^G^-Nitroarginine (L-NNA), sodium nitroprusside (SNP), and Sepiapterin were purchased from Sigma Chemicals. KN-93 was purchased from EMD Milipore. The cell permeable NO donor, DDI guanyl cyclase activator and DAR-4M AM were purchased from Enzo Life Sciences. Anti-GFP tag antibody, pluronic acid F-127, Fura-2AM and Dynabeads Protein A were purchased from Thermo Fisher Scientific. Anti-CaMKII antibody used for western blots was purchased from EMD Millipore (#07–1496) and anti-CaMKII antibody used for immunofluorescence was from LifeSpan Biosciences (LS-C100735/5122). Comparable antigen detection with the two antibodies was confirmed in pilot experiments (S1 Fig). Anti-eNOS (# 610296) used for western blotting was from B.D. Transduction Laboratories, anti-HA.11 antibody (clone 16B12, #MMS-101P) was from Covance. Antibodies directed to eNOS (#9586) (used for immunoprecipitation), GAPDH (#2178), calmodulin (#4830), phosphoThr-287 CaMKII (#12716), phosphoSer-1179 in bovine eNOS (#9571), phosphoThr-497 in bovine eNOS (pThr-495 in human) (#9574) and anti-mouse IgG-HRP (#7076S) were purchased from Cell Signaling Technology. Anti-CaMKIIN antibody was from Aviva Systems Biology (#OAAB07385). Anti-human Von Willebrand Factor antibody was from Dako (#A0082), anti-rabbit IgG-HRP was from BioRad (#1706515). Goat anti-rabbit IgG (111-066-003) and donkey anti-goat IgG (705-066-147) biotinylated antibodies and normal rabbit and normal mouse IgG were from Jackson Immuno Research. ECL was from Santa Cruz Biotechnology. Mouse-on-mouse (MOM) and Vectashield kits were purchased from Vector Labs. Rabbit anti-GFP antibody (#A6455), streptavidin conjugated fluorophores and ToPro-3 iodide were from Life Technologies. Bovine aortic endothelial cells (BAEC) and human umbilical vein endothelial cells (HUVEC) were from Cell Systems. Doxycycline (625 mg/kg) chow was from Envigo.

Human tissue samples from autopsies were procured from the University of Iowa Decedent Center in accordance with guidelines established by the University of Iowa Institutional Review Board. Upon submission of a full application, it was determined by the review board that the sample collection was exempt from federal regulations (University of Iowa IRB# 201210793). The exemption was granted on the basis that the research involved the collection or study of existing data and pathological specimens and that the information was recorded in such a manner that subjects could not be identified, directly or through identifiers linked to the subjects.

Autopsy samples were procured from subjects without a history of hypertension, diabetes, or cardiovascular disease. The absence of atherosclerosis was confirmed by gross pathology. Samples were fixed in formalin, processed and paraffin-embedded for immunohistochemistry. Some sections contained Ca^2+^ and required incubation in Decal prior to processing.

### Methods

#### Transgenic model of CaMKII inhibition in endothelial cells

All animal care and housing requirements of the National Institutes of Health Committee on Care and Use of Laboratory Animals were followed. The protocols were reviewed and specifically approved by the Iowa City VA Healthcare System and the University of Iowa Animal Care and Use Committee. To study the role of endothelial CaMKII inhibition *in vivo*, two mouse models expressing two different CaMKII small peptide inhibitors were generated. One model expressed endothelial CaMKIIN (endo-CaMKIIN) constitutively, whereas in the other model AC3-I (endo-AC3-I) expression in the endothelium was induced. The constitutive model was generated by crossing mice that express a floxed enhanced GFP sequence upstream of a stop codon followed by hemagglutinin (HA)-tagged CaMKII inhibitor peptide CaMKIIN (HA-CaMKIIN) under control of the CX-1 promoter (Tg HA-CaMKIIN) [25] with mice carrying Cre recombinase driven by the endothelial cell-specific promoter, receptor tyrosine kinase (Tek-Cre, Jackson Labs stock # 008863). All mice were in C57Bl/6 background. In the double transgene-positive progeny (endo-CaMKIIN), the floxed GFP/stop codon sequence is excised by Tie-2/Tek promoter-driven Cre recombination, allowing for HA-tagged CaMKIIN expression selectively in the endothelial layer. In contrast, GFP was expressed in all non-endothelial cells without Tek promoter activation. As expected, in littermate control mice (TekCre) that were positive for Cre but negative for the CaMKIIN transgene, no GFP was detected.

The inducible model of CaMKII inhibition was generated as follows: Tet*O*-AC3-I/eGFP mice, a kind gift of Dr. Daniel Winder that we previously described [26], were bred with mice expressing the tetracycline transactivator (tTA) under the control of the endothelial cell-specific promoter, VE-Cadherin, (FVB-Tg Cdh5.tTA, Jackson Labs stock#013585). Both the breeders and progeny were kept on a doxycycline diet (625 mg/kg, Envigo) to repress the Tet*O*. To induce AC3-I/eGFP expression in the endothelial layer, progeny that was positive for both tTA and AC3-I/eGFP transgenes (endo-AC3-I) and littermate control mice (eCdh5-tTA) that were tTA positive but AC3-I negative, were switched from the doxycycline-containing diet to normal chow for two weeks after which they were studied. All male and female mice used in experiments were between 10 and 23 weeks of age.

#### Cell culture

BAEC and HUVEC were maintained in DMEM:F12 supplemented with 10% FBS, penicillin/streptomycin, sodium pyruvate, L-glutamine, and non-essential amino acids and fed every other day. They were used between passages 7 and 11. Cells were infected at 100 moi with adenovirus expressing CaMKIIN (Ad5.CMV.CaMKIIN.HA.IRESeGFP) or control adenovirus (Ad5.CMV.Empty. IRESeGFP), plated in 60 mm dishes at a density of 400,000 cells/dish and used for further experiments after 72–96 hr [27]. Of note, both adenoviruses express eGFP and were amplified by the University of Iowa Viral Vector Core.

#### Autocamtide-2 transfection and Fura-2AM imaging

The CaMKII inhibitory peptide Autocamtide-2 (H-Lys-Lys-Ala-Leu-Arg-Arg-Gln-Glu-Ala-Val-Asp-Ala-Leu-OH, Santa Cruz Biotechnology, sc-3117) was transfected into HUVEC with the transfection reagent Chariot ^™^ (Active Motif, 30025) as recommended by the manufacturer. This approach provided CaMKII inhibition comparable to other methods as demonstrated by its effect on the downstream CaMKII signaling target eNOS (S2 Fig). HUVEC were grown in 35 mm dishes MatTek glass bottom dishes to 50% confluence and incubated with 6 μl Chariot ^™^ and 500 ng Autocamtide-2 in serum-free media for 90 min, then, growth media was added. Cells treated with transfection reagent alone served as control. 72 hr after transfection, HUVEC were labeled with FURA-2AM (2 μM in 0.02% pluronic acid, Molecular Probes, F-1201) for 30 min, followed by three washes in Hank’s buffer for 5 min and incubation for 30 min. All reactions were carried out at 37°C [25]. The cells were excited alternatively at 340 and 380 nm using a custom-built Olympus IX-81 microscope. Fluorescence signal intensity was acquired at 510 nm. The baseline was set at a F340/F380 ratio of 0.5. Real-time shifts in Fura-2 ratio fluorescence were recorded before, during and after addition of 1 μM bradykinin. Traces from 20–25 cells per condition were recorded in every experiment. For further analysis, the area under the curve for each trace from 5 sec before to 10 sec after bradykinin administration was computed using GraphPad Prism7.0 analysis software.

#### Western blotting

Cells were lysed after treatments with buffer containing 50 mM TRIS/HCl, 150 mM NaCl, 0.012 M sodium deoxycholate, 0.1% SDS, and 1% NP-40 supplemented with protease (SantaCruz Biotechnology) and phosphatase inhibitors (Thermo Fisher Scientific). Equivalent amounts of cell lysate were separated by SDS/PAGE on 4–20% TRIS/Glycine precast gels (BioRad) and transferred to PVDF membranes (Millipore). Membranes were blocked in 3% BSA/1% gelatin and incubated overnight at 4°C with primary antibodies at a dilution of 1:500 to 1:1000. Blots were washed 3 times for 10 min each with 0.05% Tween-20 in TBS and incubated for 1 hour at room temperature with the respective secondary antibodies at a dilution of 1:2000 to 1:5000. After washing, blots were developed with ECL chemiluminescent substrate (Santa Cruz Biotechnology). Blots were scanned and analyzed using Image J software.

#### Immunoprecipitation

After treatment, cells were lysed with 50 mM TRIS, 150 mM NaCl and 1% NP-40 buffer supplemented with protease and phosphatase inhibitors and 250–300 μg of cell lysate were rotated overnight at 4°C with 5 μL of anti-eNOS rabbit antibody in a final volume of 200 μL. The next day, 10 μL of Dynabeads Protein A were added to the IgG-protein complex and incubated by rotation for 4 hr at 4°C. After magnetic separation, the IgG-protein complex was eluted from the beads by boiling for 1 minute with 2X Laemmli Sample Buffer. Immunoprecipitated proteins were separated by SDS/PAGE on 4–20% TRIS/Glycine gels, transferred to PVDF membranes, and immunoblotted for eNOS and calmodulin.

#### Immunohistochemistry

Mouse thoracic aorta was fixed by immersion in 4% paraformaldehyde/PBS. After 48 hr, the aorta was cryopreserved at -80°C in Tissue Freezing Medium. Ten-μm frozen sections were cut, post-fixed on Superfrost Plus glass slides and washed in PBS. They were pre-incubated for 10 min in egg substitute, washed and blocked for 2 hr at room temperature in 5% non-fat dry milk. After washing with PBS for 10 min, the sections were incubated overnight with primary antibodies (anti-eGFP or anti-CaMKIIN) diluted 1:200 in PBS. After washes in PBS for 15 min, sections were incubated for 2 hr with biotinylated secondary antibodies diluted 1:250 in PBS. Sections were then washed with PBS for 15 min and incubated with Alexa 568-conjugated secondary. After washing in PBS, sections were counterstained with ToPro-3 iodide to visualize nuclei and mounted in Vectashield. Ten μm sections of fixed and paraffin-embedded human aorta, carotid and coronary vessels were deparaffinized and subjected to immunohistochemistry using anti-von Willebrand and CaMKII antibodies as described above. Negative controls without primary antibody were performed in every experiment. Images were captured with Zeiss LSM 710m Laser scanning microscope using the following parameters: DAPI was imaged with an excitation at 405 nm and emission filter set from 410 nm to 484 nm. CaMKII was imaged with an excitation at 488 nm and emission path from 494 nm to 572 nm and von Willebrand factor imaged with an excitation at 561 nm and emission filter set from 574 nm to 712 nm. A Plan-Apochromat 63x/1.40 Oil DIC M27 lens was used and the resolution was set to 1024 pixels in x an 1024 pixels in y, using a 8-bit encoding with a line averaging of 2. The pinhole was set to 51 μm and the dwell time to 1.58 msec. The imaging parameters were set based on the negative control samples and all images were obtained with the same parameters.

#### Nitric oxide production

NO generation in endothelial cells was measured using the cell permeable NO-sensitive dye, DAR-4M AM as described previously [28]. 60,000 endothelial cells were plated on 35 mm dishes with 25mm glass bottom inserts (MatTek plates) were infected with control or CaMKIIN- expressing adenoviruses. On day 3 post-infection, endothelial monolayers were incubated overnight with 10 μM sepiapterin, which is converted intracellularly to the eNOS cofactor tetrahydrobiopterin. The following day, cells were incubated for 30 min with 10 μM DAR-4M AM, washed. Some samples were incubated for 30 min with 100 μM L-NAME, an NO inhibitor. Baseline fluorescence of eGFP and rhodamine was captured with Zeiss LSM 510m Laser scanning microscope (eGFP excitation 488 nm, band pass 505–550 nm; DAR-4M excitation 543 nm, long pass 575 nm) at 63X magnification. Cells were then stimulated with 1 μM bradykinin and images of eGFP and rhodamine fluorescence taken again every 60 sec for 15 min. As a positive control, DAR-4M AM loaded cells were exposed to the NO donor, DD1, for 30 min. In addition, equal loading of the dye was verified by measuring rhodamine fluorescence after addition of DD1 to both Empty and CaMKIIN infected cells after bradykinin stimulation. The intensity of rhodamine and GFP fluorescence was quantified by Image J using identical settings for each frame.

#### Vasoreactivity measurements in endo-CaMKIIN mice

Vasoreactivity measurements in the thoracic aorta and third-order mesenteric arteries were performed in TekCre and endo-CaMKIIN mice described previously [29]. Mice were between 12 and 22 weeks of age. Age- and sex-matched littermates served as controls. Briefly, third-order mesenteric arteries and thoracic aorta were isolated and cleaned of adherent fat. The mesenteric arteries were cannulated onto micropipettes in an organ bath filled with Krebs buffer and pressurized to 40 mm Hg. Lumen diameter was recorded using a video camera and an electronic dimension analyzer. After equilibration for 60 min, arteries were sub-maximally pre-constricted with the thromboxane mimetic, U46619 (0.14–0.285 μM), to 50% to 60% of maximal KCl responses. After development of stable constrictions, cumulative concentration-response curves to acetylcholine (ACh), or sodium nitroprusside (SNP) were obtained. Aortic rings (3–4 mm) were suspended on wire hooks connected to a force transducer in an organ bath filled with modified Krebs (20ml, 20% O2, 5% CO2, balance N2, 37°C). Basal tension was increased over 45 min to 0.75 g prior to study. Rings were pre-constricted with U46619 (3–5 nM) to maintain a stable contraction of 50% to 60% of maximal KCl response before concentration-response curves to ACh, or SNP were recorded (PowerLab 8/30). Relaxation responses were expressed as percentage decrease in tension from pre-constriction values. To estimate NO- and prostaglandin-dependent vasoreactivity, aorta and mesenteric segments were pre-incubated for 30 min with L-NNA (10^−4^ M) or indomethacin (10^−5^ M), respectively, before stimulation with ACh or SNP.

#### Vasoreactivity measurements in C57Bl/6 mice after adenoviral transduction

Additional experiments were performed in aortic rings after transduction with adenovirus expressing CaMKIIN or control adenovirus [30]. The descending thoracic aorta of 12-week old C57Bl/6 mice was carefully excised and placed in ice-cold Krebs buffer (118.3 mM NaCl/4.7 mM KCl/2.5 mM CaCl_2_/1.2 mM KH_2_PO_4_/25 mM NaHCO_3_/1.2 mM MgSO_4_/ 11 mM glucose/0.0026 mM CaNa_2_EDTA). The aorta was cleaned of excess fat and cut transversely into 2–3 mm rings.

Aortic rings were placed overnight (16–20 hr) in EGM2 medium (Lonza, Walkersville, MD USA) in the presence of Ad5.CMV.Empty.IRESeGFP or Ad5.CMV.CaMKIIN.HA.IRESeGFP with 20x10^7^ particles per aortic ring. In pilot experiments, we determined the virus concentration that resulted in strong CaMKIIN expression in the endothelium. Transduction efficiency was assessed by eGFP fluorescence in 10-μm frozen sections of the descending thoracic aorta. Images were taken as described under “Immunohistochemistry”.

The next day, the vessels were suspended between two wire stirrups (150 mm) in a four-chamber myograph system (DMT Instruments) in 6 ml Krebs-Ringer (95% O2-5% CO_2_, pH 7.4, 37°C). The mechanical force signal was amplified, digitalized, and recorded (PowerLab 8/30). Cumulative concentration-response curves to acetylcholine (ACh, 10^−9^–10^−5^ M), or sodium nitroprusside (SNP, 10^−9^–10^−5^ M) were obtained in aortic rings after pre-contraction with phenylephrine (10^−6^ M). To estimate NO-dependent vasoreactivity, vessel segments were pre-incubated with 100 μM L-NAME for 10 min. Vasorelaxation evoked by ACh and SNP was expressed as percent relaxation, determined by calculating percentage of inhibition to the pre-constricted tension. Additional aortic rings were used for immunoblots.

#### PCR genotyping

Genomic DNA was isolated from mouse tails using DirectAmp tissue genomic DNA amplification kit (Denville Scientific, Holliston, MA). Tek-Cre genotyping was performed with the following primers: Tek-Cre forward (5′-GCG GTC TGG CAG TAA AAA CTA TC-3′), Tek-Cre reverse (5′-GTG AAA CAG CAT TGC TGT CAC TT-3′), internal control forward (5’-CTA GGC CAC AGA ATT GAA AGA TCT-3’) and internal control reverse (5’-GTA GGT GGA AAT TCT AGC ATC ATC C-3’) to amplify a 100 bp fragment of Tek-Cre and 324 bp internal control. The tail clips of endo-CaMKIIN mice were screened for GFP fluorescence. AC3-I genotyping was done with the forward primer, 5’-GCA AGG CAG TCA ACT GCC TCC TGG-3’ and the reverse primer 5’-ATG GTG AGC AAG GGC GAG GAG CTG-3’ to yield an 800 bp product. tTA genotyping was performed with the forward primer 5’-CGC TGT GGG GCA TTT TAC TTT AG-3’, reverse primer, 5’-CAT GTC CAG ATC GAA ATC GTC-3’, internal positive control forward primer 5’-CAA ATG TTG CTT GTC TGG TG-3’, and internal positive control reverse primer 5’-GTC AGT CGA GTG CAC AGT TT-3’ to yield a 450 bp tTA fragment and 200 bp internal control product.

#### qPCR

DNA-free total RNA was isolated from mouse arteries using the Micro RNeasy kit (Qiagen), and reverse transcribed using RTIII (Invitrogen). cDNA was amplified with mRNA-specific primers for CaMKIIN, AC3-I/eGFP and acidic ribosomal phosphoprotein (ARP) in Sybr Green PCR master mix (ABI) in a qPCR reaction using an ABI real-time PCR machine. CaMKIIN forward primer was 5’-ATC CTA CCC TAC GGC GAG GAC AAG -3’, CaMKIIN reverse primer was 5’-ATC CTC GAT CAC CAC TCT CTT GGC -3’, AC3-I/eGFP forward primer was 5’-TGA CCC TGA AGT TCA TCT GC-3’, AC3-I reverse was 5’- GAA GTC GTG CTG CTT CAT GT-3’, and ARP forward primer was 5’-CAT CCA GCA GGT GTT TGA CAA -3’ and ARP reverse primer was 5’-ATT GCG GAC ACC CTC TAG GAA G-3’.

#### Blood pressure measurements

Blood pressure was measured by the tail cuff method in TekCre and endo-CaMKIIN mice. The mice were age- and sex-matched and 11–22 weeks old. One week prior to the start of the experiment, mice were trained on tail cuff blood pressure equipment (BP-2000 Blood Pressure Analysis System, Visitech Systems Inc.). Thereafter, blood pressure was recorded daily for 20 min for 5 days.

To detect subtle differences, blood pressure was also monitored by radiotelemetry (PA-C10; Data Science International) in conscious, unrestrained eCdh5-tTA and endo-AC3-I mice. Mice were age- and sex-matched and 10–23 weeks old. Under ketamine (80–100 mg/kg) and xylazine (10 mg/kg) anesthesia, radiotelemetric catheters were implanted into the left common carotid artery through an anterior neck incision. The radiotelemeter transmitter was implanted subcutaneously into the left flank. After surgery, pain control was provided with flunixin meglumine and meloxicam. Six days after surgery, arterial blood pressure, heart rate and activity levels were recorded at 500 Hz for 10-sec intervals every 10 min over a total period of 48 hr. The following day, mice were switched from doxycycline-containing chow to normal chow to induce endothelial AC3-I expression. After 14 days on the normal chow, arterial blood pressure, heart rate, and activity levels were recorded as above. The averages of 48-hr recordings were calculated in each mouse.

#### Plasma nitric oxide determination

Blood from the left ventricle of eCdh5-tTA and endo-AC3-I mice that had been on regular chow for at least two weeks was collected in BD Microtainer tubes. Plasma was separated following centrifugation at 9300 x g for 5 min and immediately frozen at -80°C. Total reducible NO content (NO_x_) in thawed samples of plasma was determined using a Sievers NOA 280i Nitric Oxide Analyzer (GE Analytical Instruments, Boulder, CO) and includes NO released from nitrosothiols, nitrite and nitrate [31]. Reduction and subsequent release of NO was enabled by refluxing with argon gas in the presence of the reducing agent, 1% w/v vanadium (II) chloride in1M HCl at 90°C as per the manufacturer’s guidelines. The amount of NO release was determined by comparison to standard curves derived from known concentrations of NaNO_3_ in water.

#### Statistical analyses

Data are expressed as mean SEM and analyzed by with GraphPad Prism 7.0 software using 2-tailed Unpaired Student’s t-test (Fig 2C and 2F; Fig 3A, 3B and 3G; Fig 6B and 6D and S2 Fig), 2-way ANOVA followed by Bonferroni’s multiple comparison test (Fig 3C–3F), 2-way ANOVA followed by Tukey’s multiple comparison test (Figs 4A–4D, 7D, 8), and 2-way ANOVA followed by Sidak’s multiple comparison test (Figs 5E, 7C and 7F). S4 was analyzed by one-way ANOVA followed by Tukey’s Tukey’s multiple comparison test. A p-value < 0.05 was considered significant.

## Results

### Models of CaMKII inhibition in endothelium

**Fig 1:**
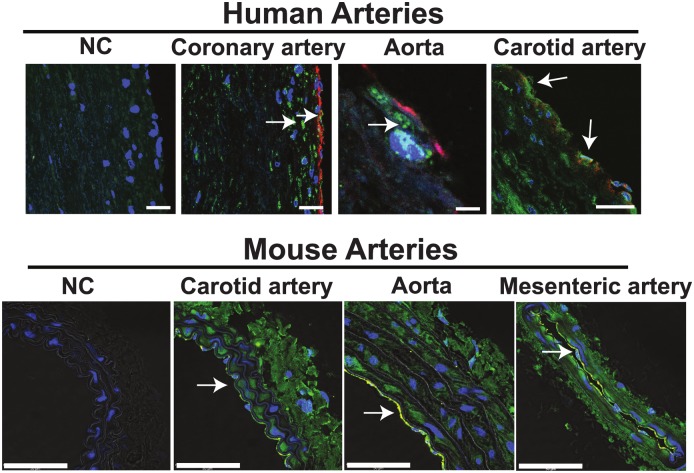
CaMKII is abundantly expressed in endothelium of different arterial beds. Immunofluorescence of CaMKII. Red: von Willebrand factor (endothelium); green: CaMKII; yellow: merged von Willebrand factor and CaMKII; blue: DAPI (nuclei). Arrows denote CaMKII expression. NC: negative control without primary antibody. Immunofluorescence in three arterial beds in human autopsy samples (Scale bars: 25 μm coronary and carotid artery, 5 μm aorta) and in three arterial beds in murine samples. (Scale bars: 50 μm).

We first examined the expression of CaMKII in the human and murine arterial wall by immunofluorescence. In a variety of vascular beds, CaMKII was strongly detected in endothelium and, as expected, in medial smooth muscle cells (Fig 1). These results prompted us to engineer novel genetic mouse models to selectively inhibit CaMKII activity in endothelium by transgenic expression of the specific CaMKII inhibitor peptides, CaMKIIN [23] or AC3-I [16](referred to as endo-CaMKIIN or endo-AC3-I mice) analogous to previously published models of CaMKII inhibition in other cell types and tissues [8, 25, 26].

**Fig 2:**
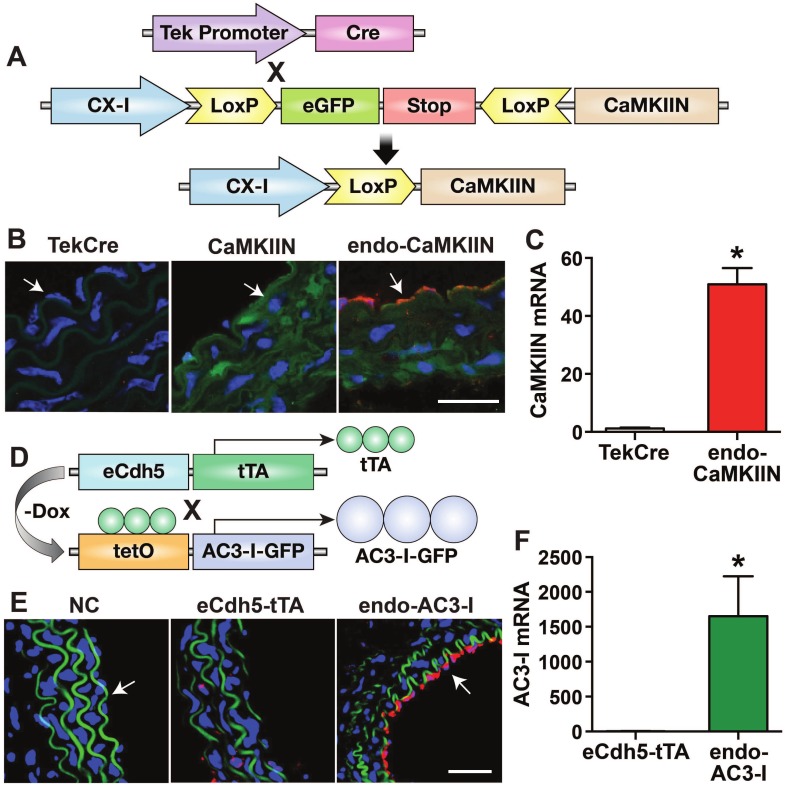
Mouse models of endothelial CaMKII inhibition. **A.** Schematic of endo-CaMKIIN mouse model. **B.** Immunofluorescent detection of HA-tagged CaMKIIN in aorta from littermate controls (TekCre mice), CaMKIIN mice before TekCre recombination, and in endo-CaMKIIN mice (after Cre recombination). Red: CaMKIIN; green: eGFP (expressed before Cre recombination); blue: nuclei. **C.** Analysis of CaMKIIN mRNA levels by qRT-PCR. **D.** Schematic of endo-AC3-I mouse model. Expression of AC3-I occurs upon doxycycline (Dox) withdrawal. **E.** Immunofluorescent detection of eGFP-tagged AC3-I in littermate controls (eCdh5-tTA mice) and endo-AC3-I mice. Red: GFP; green: autofluorescence of elastin fibers; blue: nuclei. NC: negative control without primary antibody. **F.** Analysis of AC3-I mRNA levels by qRT-PCR. In **B, E,** Scale bars: 50 μm; arrows denote endothelium. Data in **C** and **F** are normalized to ARP and results expressed as mean ± SEM, n = 3 and 6 mice/group in C and F, respectively. *p<0.05 vs control.

In a constitutive model of endothelial CaMKII inhibition with CaMKIIN (Fig 2A), we confirmed that transgene is expressed in endothelium after Cre recombination only (Fig 2B). In this model, endothelial-specific transgene expression is achieved via recombination with Cre driven by a Tek promoter. Compared to TekCre mice that served as WT littermate controls, a 50-fold increase in transgene mRNA levels was detected in aortas of endo-CaMKIIN mice by qrtPCR (Fig 2C).

Since chronic CaMKII inhibition may induce compensatory mechanisms that counteract effects of the inhibitor, and transgene expression in Tek-driven Cre models also occurs in bone-marrow derived cells, we opted to engineer a second model of inducible CaMKII inhibition. In this model, the inhibitor peptide AC3-I fused to GFP is expressed under the control of the cadherin-5 (eCdh5) promoter upon doxycycline withdrawal (Fig 2D). eCdh5-tTA mice served as WT littermate controls. Based on our prior experience with this model, AC3-I expression was induced by doxycycline withdrawal for 10–14 days prior to the start of the experimental protocols. At this time point, detection of the AC3-I-GFP fusion protein by immunofluorescence in the aorta confirmed its localization to the endothelium (Fig 2E). Moreover, transgene mRNA levels were increased more than 1000-fold over control (Fig 2F). AC3-I-GFP fusion protein expression did not significantly affect the CaMKII protein levels in the aorta (S3 Fig).

### CaMKII inhibition does not affect baseline blood pressure or plasma NO levels

**Fig 3:**
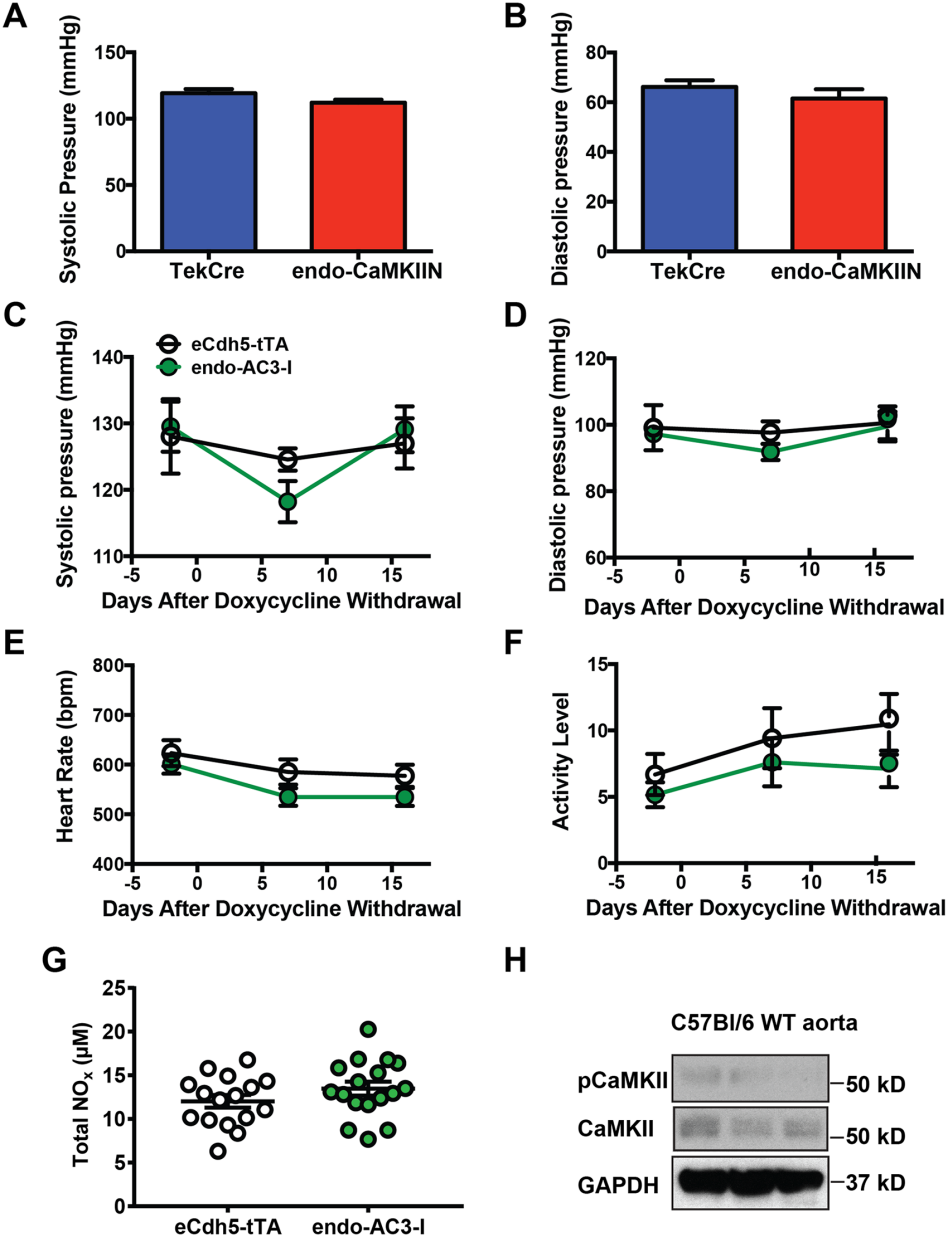
Endothelial CaMKII inhibition does not affect baseline blood pressure or plasma NO levels. **A, B.** Basal systolic (**A**) and diastolic (**B**) blood pressures by tail cuff method in control and endo-CaMKIIN mice (n = 7–9 mice/group). **C, D.** Basal systolic (**C**) and diastolic (**D**) blood pressures by radiotelemetry in control and endo-AC3-I mice at indicated days after doxycycline withdrawal (n = 6–9 mice/group). **E, F.** Heart rate (**E**) and activity levels (**F**) in control and endo-AC3-I mice (n = 6–9 mice/group). **G**. Levels of total reducible NO (NOx), that includes nitrites and nitrates, in plasma of endo-AC3-I mice and littermate controls using a Sievers Nitric Oxide Analyzer NOA280i (n = 16–17 mice/group). **H.** Immunoblot for active CaMKII pThr-287 and total CaMKII in aortas of C57Bl/6 WT mice.

Since NO production is a key mechanism by which the endothelium impacts vascular function and previous reports have yielded contradictory results on CaMKII-dependent eNOS activation [18, 19], we interrogated systolic and diastolic blood pressure and heart rate in endothelial CaMKIIN- or AC3-I-expressing mice. No difference in systolic or diastolic blood pressure was observed between control and endo-CaMKIIN mice with constitutive CaMKIIN expression by tail-cuff measurements (Fig 3A and 3B). To detect subtle blood pressure differences in an acute model of CaMKII inhibition, we performed blood pressure measurements by telemetry in endo-AC3-I mice. Like in endo-CaMKIIN mice with chronic CaMKII inhibition, systolic and diastolic blood pressures were unaltered in endo-AC3-I mice before and after induction of transgene expression by withdrawal of doxycycline compared to control mice on the same regimen (Fig 3C and 3D). Neither heart rate nor activity levels were altered by endothelial AC3-I expression (Fig 3E and 3F).

Next, we tested whether CaMKII inhibition affects serum NO in the inducible model of CaMKII inhibition (endo-AC3-I). As implied by the results of the blood pressure measurements, no significant differences in plasma NO, as determined by measuring total reducible NO (nitrites and nitrates) were detected with a Sievers Nitric Oxide Analyzer (Fig 3G). These data imply that CaMKII in endothelial cells does not control baseline blood pressure via NO.

We next assessed CaMKII activity in the arterial wall at baseline conditions by immunoblots for CaMKII autophosphorylation at Thr-287. In aortic lysates from WT mice, we detected low levels of active CaMKII (Fig 3H), suggesting that baseline CaMKII activity in the aortic wall is minimal.

### Endothelial CaMKII inhibition does not decrease endothelium-dependent vasodilation

**Fig 4:**
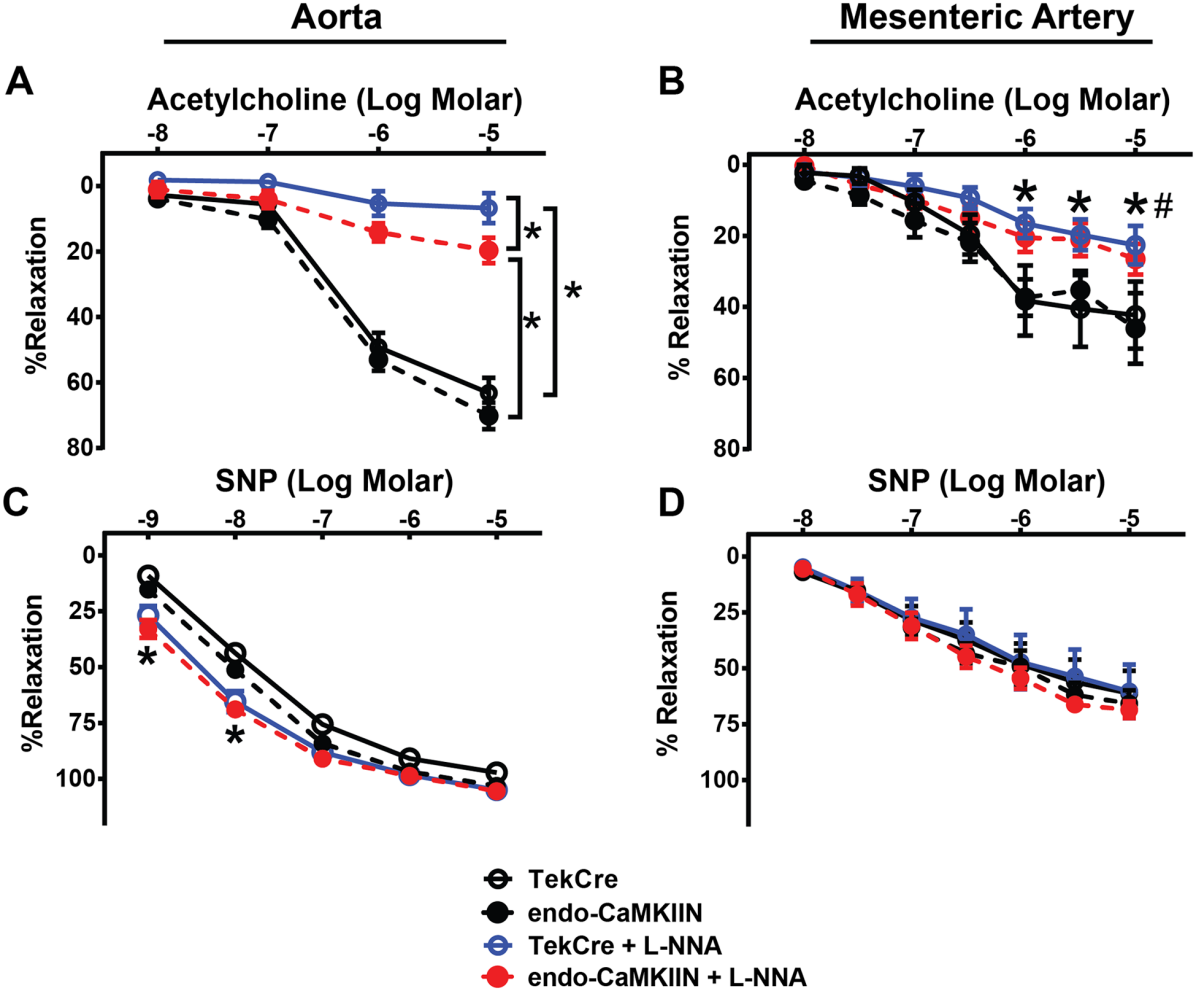
Inhibition of endothelial CaMKII does not affect vasorelaxation. **A.** Vascular relaxation in response to acetylcholine (ACh) in the presence or absence of the NOS inhibitor, L-NNA, was measured in pre-constricted aorta segments isolated from TekCre or endo-CaMKIIN mice (n = 11 TekCre and n = 9 endo-CaMKIIN mice/group). * p<0.05. **B**. Vascular relaxation in response to SNP in the presence or absence of L-NNA was measured in pre-constricted aorta segments from TekCre or endo-CaMKIIN mice (n = 10 TekCre and n = 8 endo-CaMKIIN mice/group). * p<0.05 vs. absence of L-NNA. **C.** Vascular relaxation in response to ACh in the presence or absence of L-NNA was measured in pre-constricted mesenteric arterial segments isolated from TekCre or endo-CaMKIIN mice (n = 7–8 TekCre and n = 7–8 endo-CaMKIIN mice/group). **D.** Vascular relaxation in response to SNP in the presence or absence of L-NNA was measured in pre-constricted mesenteric arterial segments isolated from control or endo-CaMKIIN mice (n = 7–8 TekCre and n = 6–8 endo-CaMKIIN mice/group). *p<0.05 vs. absence of L-NNA.

Previous evidence suggests that CaMKII modulates vasodilation secondary to inducing eNOS activation [15]. However, this study was performed with the pharmacological inhibitor KN-93 that has known CaMKII-independent effects and could alter vasomotor tone via alternative mechanisms [16]. Therefore, we examined vasoreactivity in response to increasing concentrations of acetylcholine in pre-constricted aorta and mesenteric arterial segments isolated from control and endo-CaMKIIN mice. Contrary to previous data with KN-93, vasodilation in response to acetylcholine was unaltered in both aorta and mesenteric segments from endo-CaMKIIN mice compared to control mice (Fig 4A) [15]. As expected, the eNOS inhibitor, L-NNA, significantly inhibited acetylcholine-stimulated vasorelaxation in the aorta and mesenteric arteries. However, the inhibition was significantly weaker in endo-CaMKIIN aortic segments suggesting that constitutive CaMKII inhibition induces compensatory NO-independent vasodilation. No significant differences between the two genotypes were observed after application of L-NNA in the mesenteric arterial segments (Fig 4B). There was no impairment in the smooth muscle cell contractility as the NO donor, SNP, equally induced relaxation in aorta and mesenteric artery from both groups of mice (Fig 4C and 4D).

**Fig 5:**
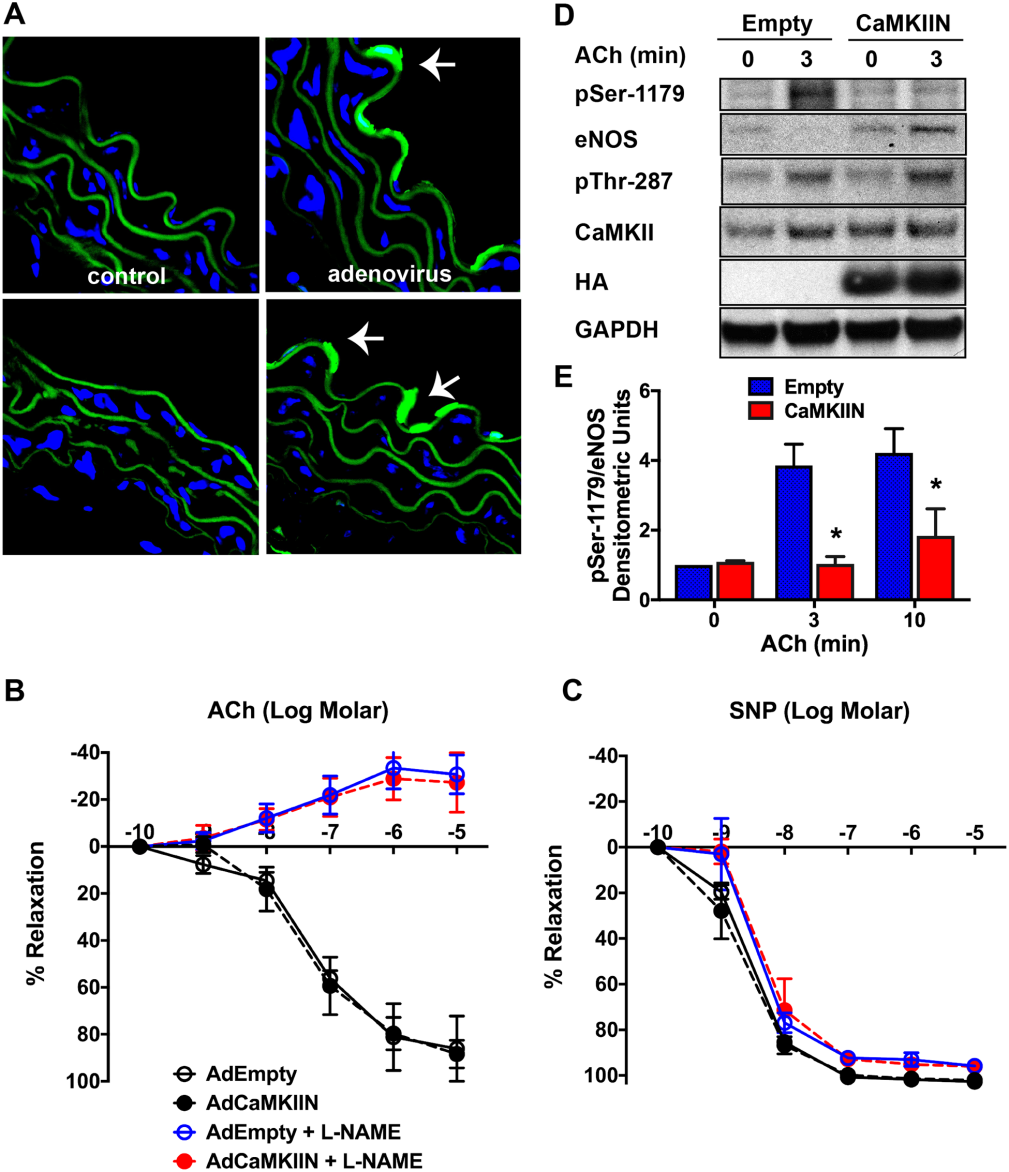
Ex vivo CaMKIIN delivery does not alter ACh-dependent vasorelaxation. **A**. eGFP expression in aortic rings of C57Bl/6 mice that were incubated with 20x10^7^ particles of adenovirus expressing CaMKIIN (Ad5.CMV.IRES.CaMKIIN.HA) or no virus for 16–20 hr. Green: GFP, Blue: DAPI (nuclei). Arrow denotes endothelium. **B, C.** Vascular relaxation in response to Ach (**B**) or SNP (**C**) in aortic rings of C57Bl/6 mice after treatment with 20x10^7^ particles of adenovirus expressing CaMKIIN or control adenovirus (Empty) for 16–20 hr. The same conditions were used in A, B, C. Some aortic rings were preincubated with 100 μM L-NAME for 10 min. **D.** Immunoblots for eNOS pSer-1179, eNOS, CaMKII pThr-497, CaMKII, GAPDH and HA were performed in aortic rings that were treated as indicated. **E.** Densitometric quantitation of the fold-increase in eNOS pSer-1179 relative to total eNOS. Data are the average of three independent experiments; *p<0.05 vs. Empty.

To confirm these findings, we performed vasoreactivity studies in aortic rings from C57Bl/6 mice that were incubated ex vivo with adenovirus that expresses the inhibitor peptide CaMKIIN. Despite robust transgene expression, no differences in vasodilation to ACh or SNP were detected (Fig 5A–5C). We also assessed eNOS phosphorylation at Ser-1179 in aortic rings that were exposed to ACh. Here, we detected a significant decrease in active eNOS in aortic rings incubated with adenovirus expressing CaMKIIN (Fig 5D). ACh treatment increased CaMKII autophosphorylation at Thr-287 in both groups. This is not surprising since the inhibitor peptide CaMKIIN blocks substrate binding, but does not interfere with CaMKII autophosphorylation [32].

### CaMKII is activated in response to bradykinin and promotes intracellular Ca^2+^ increases

**Fig 6:**
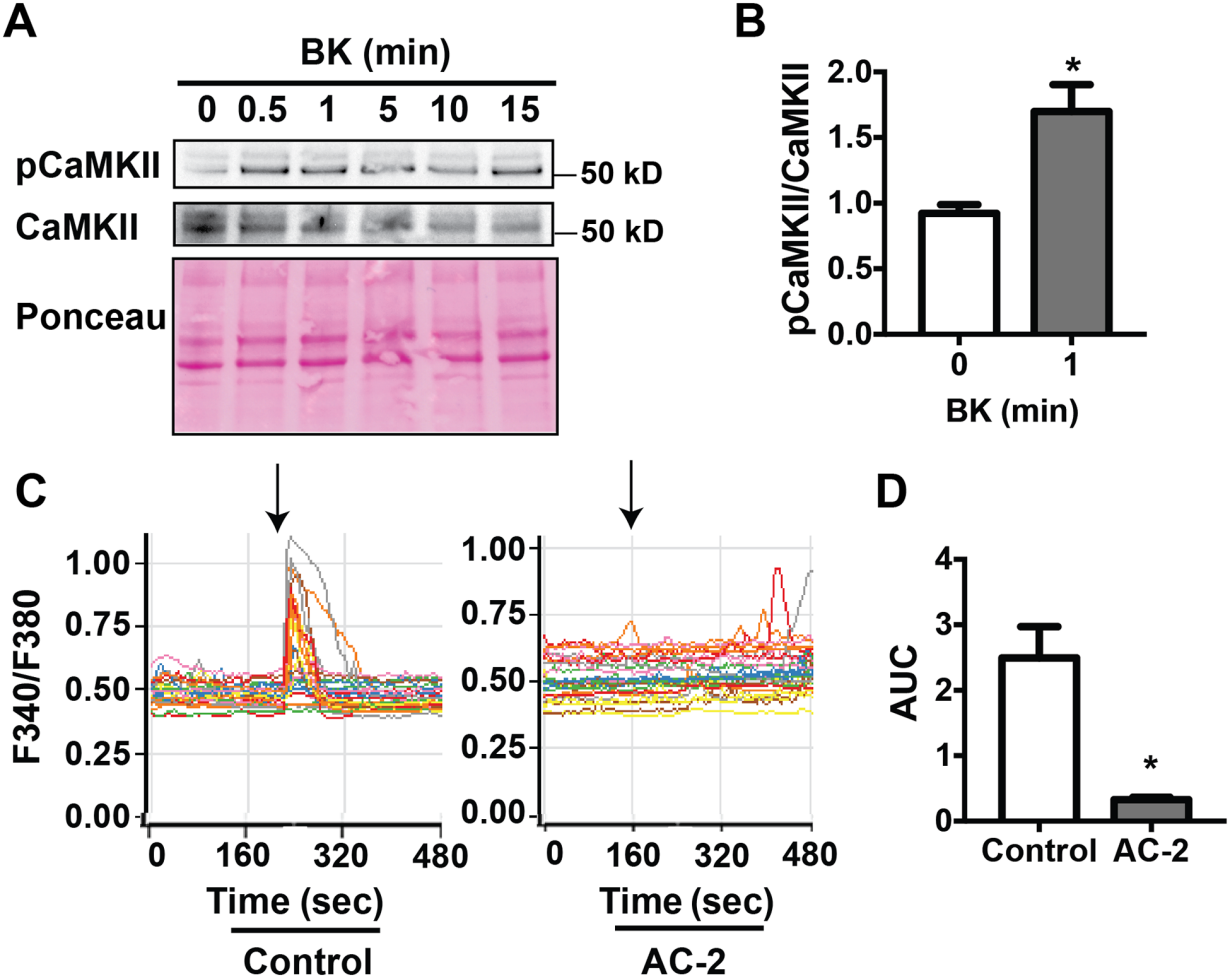
Bradykinin induces rapid CaMKII activation and Ca^2+^ influx *in vitro*. **A**. Representative immunoblots of phosphoThr-287 CaMKII (pCaMKII) and total CaMKII in 1 μM bradykinin (BK)-treated BAEC. **B**. Densitometric quantitation of the fold-increase in pCaMKII relative to total CaMKII. Data are the average of 4 independent experiments; *p<0.05 vs. 0 min time point. **C**. Representative tracings of BK-induced changes in Fura-2AM ratios in control HUVEC or HUVEC transfected with the CaMKII-specific inhibitory peptide autocamtide-2 (AC-2). Arrows indicate the time point when BK (1 μM) was administered. Each tracing represents a single cell. **D**. Areas under the curves from 5 sec before to 10 sec after BK administration. n = 4 independent experiments, *p<0.05 vs. control.

Previous studies in cultured endothelial cells *in vitro* were primarily performed with KN-93, a pharmacologic inhibitor that has known CaMKII-independent effects [14, 18–20]. These studies yielded conflicting data on CaMKII as regulator of eNOS activation and NO production [18, 19]. Our approach to inhibit CaMKII with the CaMKIIN inhibitor peptide, which has no known off-targeting effects, is more likely to provide more conclusive evidence on CaMKII regulation of eNOS function. First, we confirmed that treatment of BAEC with bradykinin resulted in rapid phosphorylation of CaMKII that was sustained for up to 15 min (Fig 6A and 6B).

Because of previous reports on CaMKII as regulator of cytosolic Ca^2+^ in endothelial cells [12], we next measured intracellular Ca^2+^ in Fura-2AM-loaded cells transfected with control peptide or the CaMKII inhibitory peptide, Autocamtide-2. This peptide was chosen because our adenoviral CaMKIIN construct co-expresses GFP, which precludes the use of Fura-2AM. A two-fold increase in intracellular Ca^2+^ concentration was observed within seconds of bradykinin addition to control cells, whereas CaMKII inhibition prevented the agonist-induced rapid rise in intracellular Ca^2+^ levels (Fig 6C and 6D).

### CaMKII modulates eNOS activation

**Fig 7:**
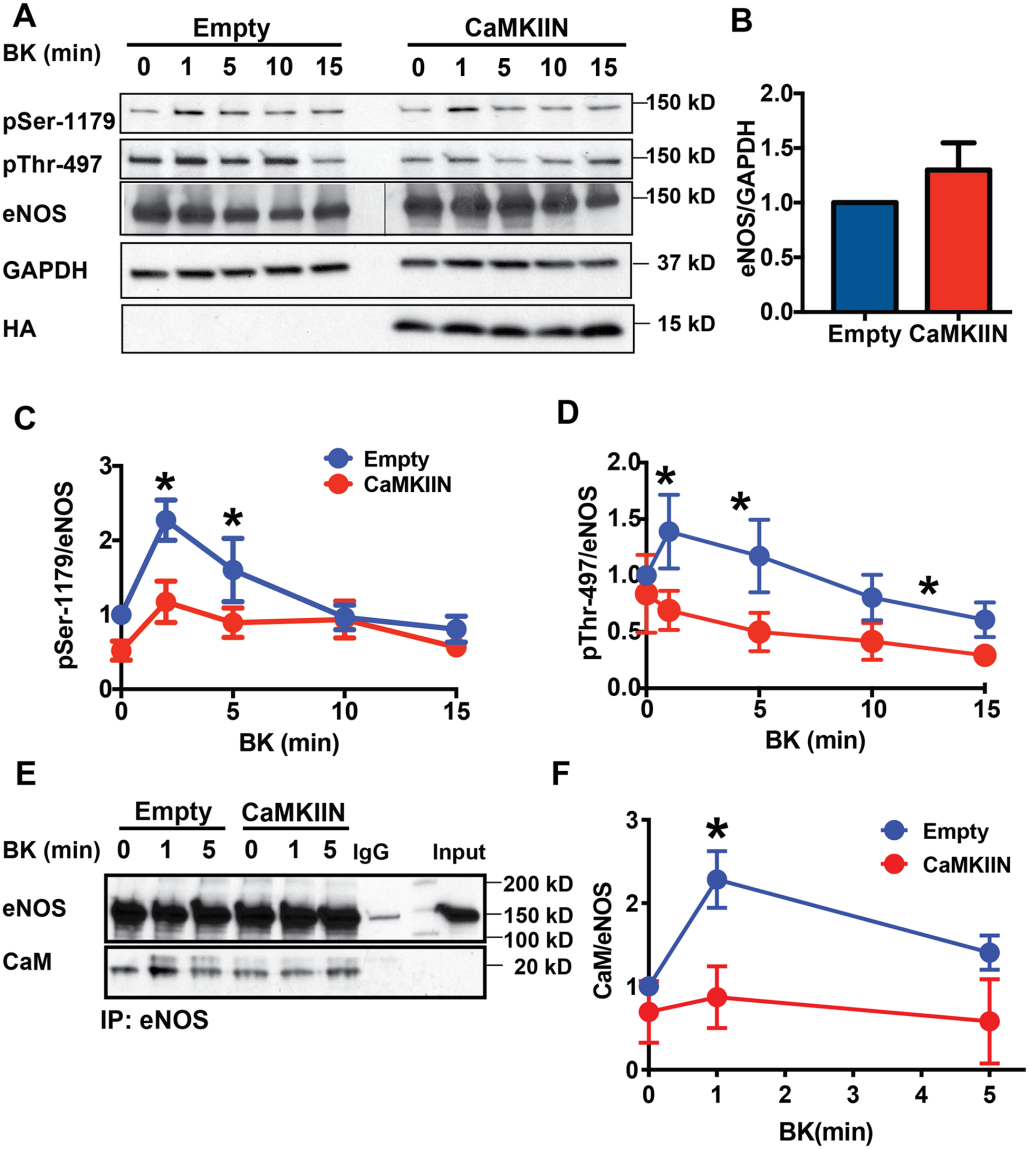
Acute inhibition of CaMKII attenuates eNOS activation *in vitro*. **A**. eNOS phosphorylation at Ser-1179 and Thr-497 following bradykinin (BK) treatment in BAEC infected with adenovirus expressing CaMKIIN or control adenovirus (Empty). Please note that in the eNOS immunoblot a molecular weight standard was loaded in an additional lane in between Empty and CaMKIIN treatment groups. **B**. Densitometric quantification of eNOS in BAEC infected with adenovirus expressing CaMKIIN or control adenovirus (Empty) (n = 8 independent experiments). **C.** Densitometric quantification of pSer-1179 immunoblots normalized total eNOS (n = 7 representative experiments); *p<0.05 vs. CaMKIIN at respective time point. **D**. Densitometric analyses of pThr-497 immunoblots normalized total eNOS (n = 7 representative experiments); *p<0.05 vs. CaMKIIN at respective time point. **E**. Calmodulin (CaM) binding to eNOS estimated by co-immunoprecipitation in cells stimulated with BK. **F**. Densitometric analyses of CaM binding to eNOS relative to total eNOS (n = 3 independent experiments), *p<0.05 vs. CaMKIIN at respective time point.

eNOS can be activated by both Ca^2+^-dependent as well as -independent mechanisms [33]. Its activation following stimulation by most agonists, such as bradykinin acting through the GPCR, is largely dependent on the rise in intracellular Ca^2+^ [18]. As CaMKII inhibition prevented bradykinin-induced Ca^2+^ influx, we investigated its effect on eNOS activation. eNOS activation was defined as an increase in Ser-1179 phosphorylation, Thr-497 dephosphorylation and Ca^2+^/CaM binding following bradykinin stimulation. Phosphorylation on Ser-1179 residue peaked after 1 minute of bradykinin treatment and rapidly declined to baseline levels in control cells (Fig 7A and 7C). CaMKIIN attenuated this increase without affecting eNOS protein expression (Fig 7B and 7C). Bradykinin also affected phosphorylation of Thr-497 in control but not in CaMKIIN-expressing cells (Fig 7C). This is in contrast to previously reported data with KN-93 [18]. Next, we examined binding to Ca^2+^/CaM, which occurs after eNOS dephosphorylation at Thr-497. eNOS was immunoprecipitated from control and CaMKIIN-expressing endothelial cells incubated with bradykinin followed by immunoblotting for CaM. As expected, bradykinin induced Ca^2+^/CaM association with eNOS in control cells; however, the co-immunoprecipitation of CaM was attenuated in CaMKIIN-expressing cells (Fig 7E and 7F).

### CaMKII promotes endothelial NO production in vitro

**Fig 8:**
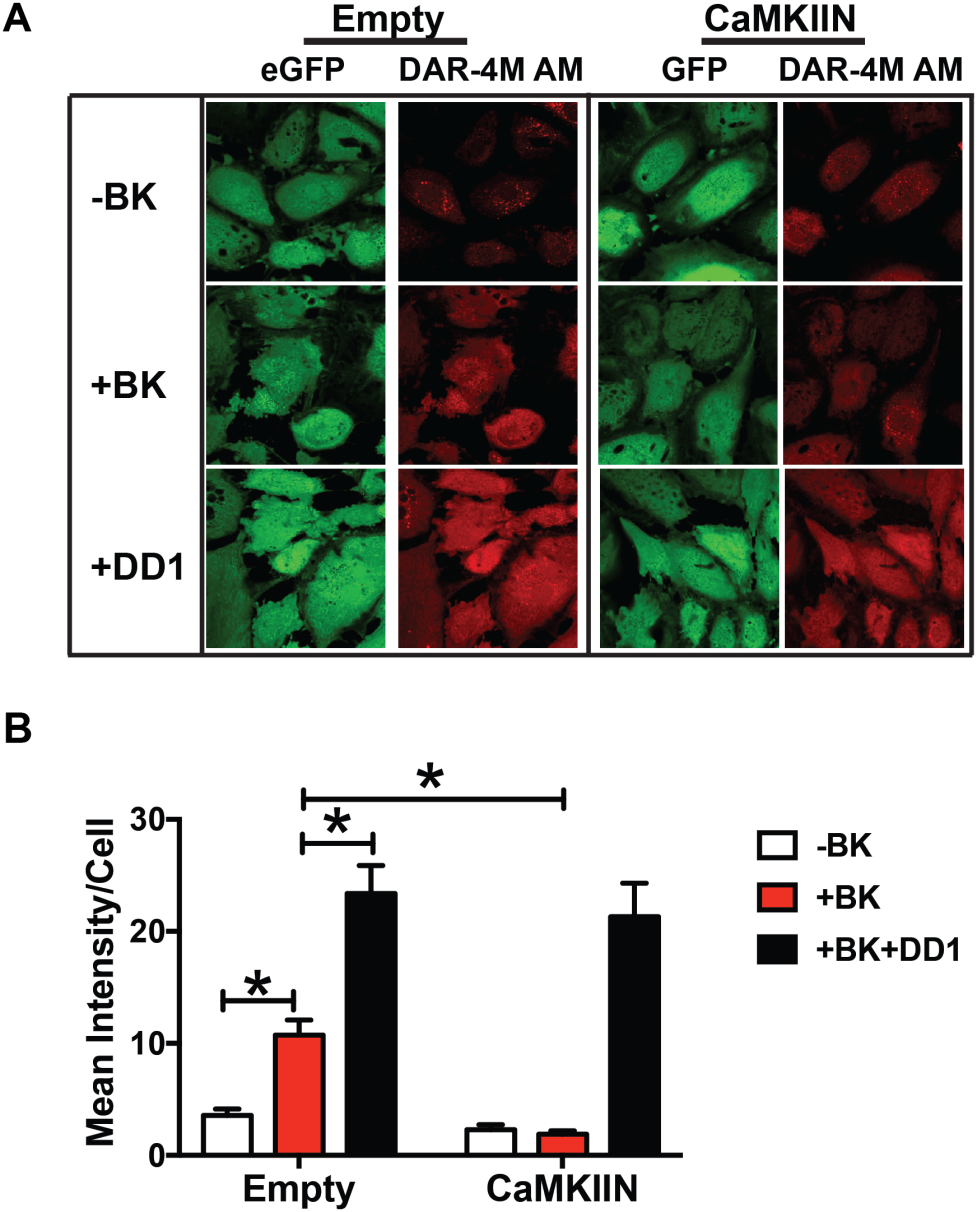
Inhibition of CaMKII abrogates bradykinin-induced NO production *in vitro*. **A**. NO-sensitive DAR-4M FM fluorescence in BAEC infected with adenovirus expressing CaMKIIN or control adenovirus (Empty) with and without treatment with 1μM bradykinin (BK). Green: eGFP; red: DAR-4M FM. As a positive control, cells were incubated with 10 μM of the NO donor DD1 after exposure to BK. **B**. Densitometric analyses of the intensity of the DAR-4M FM signal. Data are the average of 3 independent experiments. * p<0.05 vs. CaMKIIN.

To understand the net effect of these findings, we tested whether CaMKII inhibition affects NO production in bradykinin-stimulated endothelial cells infected with either empty or CaMKIIN virus. As expected, bradykinin significantly induced NO production in control cells but little to no response to bradykinin was detected in CaMKIIN-expressing cells (Fig 8 and S4 Fig). This difference was not due to inadequate labeling of CaMKIIN-expressing cells with DAR-4M FM probe as addition of the exogenous NO donor DD1 resulted in an equally robust signal in CaMKIIN-expressing and control cells.

## Discussion

CaMKII has been extensively studied in excitable cells such as neuronal cells and cardiac myocytes. However, its role in the vasculature is less well investigated. Our current understanding is limited to its actions in the smooth muscle cell layer of the vascular wall [6, 8, 25, 34] but its functions, particularly in the endothelium *in vivo* are incompletely understood. Most of our knowledge of CaMKII is based on the use of non-selective inhibitors such as KN-93, which have known off-target effects on ion channels and other kinases [16]. Other approaches to manipulate CaMKII activity by for example siRNA or inhibitory peptides such as Autocamtide-2 are restricted to cultured endothelial cells, which, depending on their passage number, can differ significantly from their *in vivo* counterparts. Thus, our understanding of the role of CaMKII in endothelium has been severely impaired by a lack of suitable tools that translate both *in vitro* and *in vivo*. Thus, to overcome these obstacles, we engineered novel mouse models that express highly selective and potent inhibitors of CaMKII in the endothelium.

NO is well accepted to control vascular tone and blood pressure *in vivo* [35], and some evidence suggests that CaMKII regulates NO production [15, 18]. Thus, we used our novel *in vivo* models to determine the role of endothelial CaMKII in hemodynamic parameters. We demonstrate that the activity of CaMKII in the aortic wall is low at baseline. These findings correspond to previous results in mesenteric arteries [36]. Thus, inhibition of endothelial CaMKII had no impact on baseline plasma nitric oxide levels and blood pressure *in vivo*. Moreover, no difference in eNOS-dependent vasodilation was found. However, CaMKII inhibition with CaMKIIN modulated phosphorylation at the regulatory site Ser-1179 in aortic rings and cultured aortic endothelial cells.

While total body deletion of eNOS in mice impairs vasodilation to ACh and increases basal mean blood pressure by 30–50 mmHg [37, 38], several studies have demonstrated that abolishing eNOS regulation at the phosphorylation site Ser-1179 only has a mild effect on vasodilation. First, in carotid arteries of mice that express the inactive eNOS S1179A transgene on an eNOS^-/-^ background, some recovery of vasodilation to ACh is observed, whereas expression of the constitutively active S1179D does not normalize vasodilation to the level seen in WT mice [39]. Furthermore, blood pressure in the two mouse strains is similar and not different from eNOS^-/-^ mice. Similarly, adenoviral expression of the inactive S1179A eNOS mutant in aortic rings only mildly affects ACh-dependent vasodilation compared WT eNOS [40]. These data demonstrate that while extensively studied *in vitro* and in other *in vivo* readouts of eNOS function, eNOS phosphorylation at these sites is not a determinant of blood pressure, potentially because the phosphorylation is short-lived.

Our study is not the first to suggest that the *in vivo* phenotypes and *in vitro* eNOS phosphorylation do not completely align. The kinase Akt-1 has been extensively studied as a regulator of eNOS phosphorylation at Ser-1179 [41, 42]. However, in two studies with Akt-1^-/-^ mice, no differences in blood pressure and/or vasodilation to ACh were found [43, 44]. In another study that used the approach of adenoviral delivery of dominant negative Akt-1 in WT aortas, vasodilation is only mildly reduced as compared to overexpression of WT Akt-1 [45]. The lack of the *in vivo* phenotype with endothelial CaMKII inhibitor mice is not likely attributed to insufficient inhibition of CaMKII given that we observed robust endothelial CaMKIIN and AC3-I expression equivalent to or greater than previously reported in other models of cell type-specific CaMKII inhibition [5, 8, 26]. This is further supported by the immunoblots in aortic rings that demonstrate an effect on eNOS phosphorylation similar to our *in vitro* findings (Figs 5 and 7). Our ex vivo vasoreactivity experiments in aortic rings transduced with CaMKIIN rule out that the adenoviral expression of CaMKIIN causes unanticipated off-target effects.

Constitutive inhibition of endothelial CaMKII *in vivo* mildly, but significantly altered the residual vasorelaxation to ACh after eNOS inhibition. We interpret this finding as evidence that chronic CaMKII inhibition stimulates compensatory eNOS-independent mechanisms that promote vasodilation such as EDRF and PGE2 [46, 47]. These data also support that in our transgenic model, CaMKIIN is active in the vascular endothelium.

Many agonists, such as bradykinin, induce eNOS activation by increasing Ca^2+^ influx [33, 48]. Our data reveal that inhibition of CaMKII almost completely abolished bradykinin-induced Ca^2+^ influx. Numerous studies have demonstrated that CaMKII plays an important role in ER Ca^2+^ release and capacitative Ca^2+^ entry following agonist stimulation of endothelial cells [12]. Therefore, CaMKII likely modulates eNOS activation *in vitro* via Ca^2+^ responses after agonist stimulation. Supporting this, the formation of a complex between the transient receptor potential (TRPV1), Akt, CaMKII and eNOS results in phosphorylation of eNOS following Ca^2+^ influx [49, 50]. Our observations are consistent with this given that inhibition of CaMKII prevented Ca^2+^ entry and subsequently attenuated eNOS phosphorylation on Ser-1179. Similarly, Fleming and colleagues demonstrated increased binding of CaMKII to eNOS following bradykinin stimulation of porcine aortic endothelial cells and suggested that CaMKII directly phosphorylates eNOS on Ser-1179 [18]. By contrast, in a second study, KN-93 did not block bradykinin-mediated eNOS phosphorylation [19]. Our data provide further substantiation for CaMKII regulation of eNOS phosphorylation on Ser-1179.

In summary, our data identify CaMKII as a regulator of eNOS phosphorylation and NO production in endothelium *in vitro*. This is agreement with other studies in smooth muscle and cardiomyocytes where CaMKII activation *in vivo* under baseline physiological conditions is low, whereas pathological activation of CaMKII has been documented in numerous disease states, including diabetes and hypertension [8, 51]. Therefore, it is highly probable that CaMKII contributes to endothelial-dependent vascular disease, which can be queried using our novel models of endothelial-specific CaMKII inhibition *in vivo*.

## Supporting information

S1 Fig CaMKII detection. Immunoblots for total CaMKII protein in lysates from aortas of C57Bl/6 mice, BAEC and HEK cells infected with an adenovirus expressing CaMKIIδ for 72 hr and blotted with an anti-CaMKII antibody from EMD Millipore used for immunoblots in Figs 3, 5, 6 and 7 (#07–1496) and an anti-CaMKII antibody from LifeSpan Biosciences that used for immunofluorescence in Fig 1 (LS-C100735/5122).
(PDF)

S2 Fig Approaches to inhibit CaMKII and their effect on eNOS Ser-1179. BAEC were treated with KN-93 for 2 hr or transfected with the CaMKII inhibitory protein Autocamtide-2 using the the transfection reagent Chariot for 48 hr. Treatment with bradykinin was performed after serum starvation for 24 hr. Representative immunoblots for eNOS pSer-1179 and eNOS. These approaches resulted in eNOS inhibition comparable to inhibition with CaMKIIN as shown in Fig 7.
(PDF)

S3 Fig CaMKII expression after CaMKII inhibition with AC3-I. (A) Immunoblots for total CaMKII protein in aortas of eCdh5-tTA control and endo-AC3-I mice (lysates from one mouse per lane). (B) Quantiifcation of data in (A). Mean±SEM, n = 10 mice/group.
(PDF)

S4 Fig eNOS inhibition with L-NAME abrogates NO production in endothelial cells. NO-sensitive DAR-4M FM fluorescence in bovine aortic endothelial cells infected with control adenovirus Ad5.CMV.IRES.eGFP.Empty (control adenovirus expresses eGFP). Treatment with 1μM bradykinin. Additional samples were pretreated with 100μM L-NAME for 30 minutes. Green: eGFP; red: DAR-4M FM. B. Densitometric analyses of the DAR-4M FM signal. Data are the average of 3 independent experiments. * p<0.05 vs.—BK.
(PDF)
