# Supplementary figures and images for: Endothelial CaMKII as a regulator of eNOS activity and NO-mediated vasoreactivity
Source: PLoS One. 2017 Oct 23;12(10):e0186311. doi: 10.1371/journal.pone.0186311 (PMC5653296; doi:10.1371/journal.pone.0186311)

**S1 Fig**

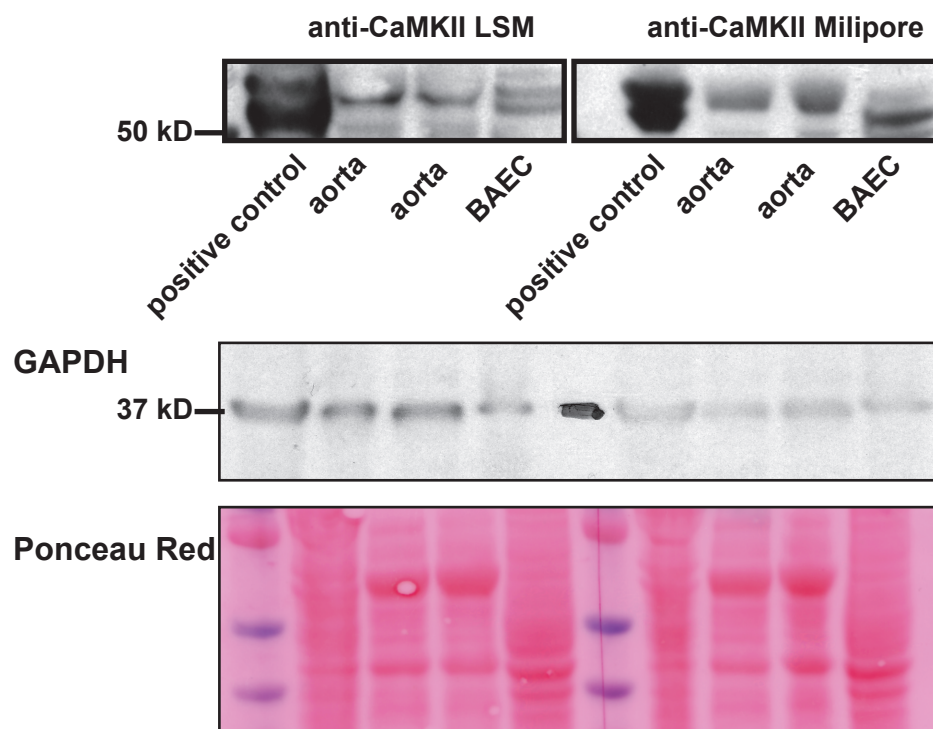

Supplement: S1 Fig — Immunoblots for total CaMKII protein in lysates from aortas of C57Bl/6 mice, BAEC and HEK cells infected with an adenovirus expressing CaMKIIδ for 72 hr and blotted with an anti-CaMKII antibody from EMD Millipore used for immunoblots in Figs 3, 5, 6 and 7 (#07–1496) and an anti-CaMKII antibody from LifeSpan Biosciences that used for immunofluorescence in Fig 1 (LS-C100735/5122). (PDF) [file pone.0186311.s001.pdf]

S2 Fig

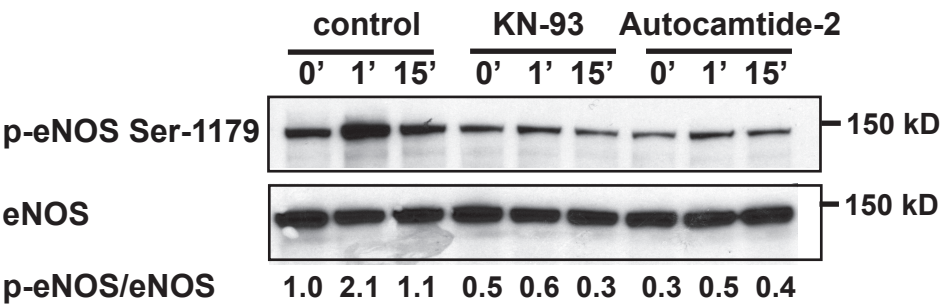

Supplement: S2 Fig — BAEC were treated with KN-93 for 2 hr or transfected with the CaMKII inhibitory protein Autocamtide-2 using the the transfection reagent Chariot for 48 hr. Treatment with bradykinin was performed after serum starvation for 24 hr. Representative immunoblots for eNOS pSer-1179 and eNOS. These approaches resulted in eNOS inhibition comparable to inhibition with CaMKIIN as shown in Fig 7. (PDF) [file pone.0186311.s002.pdf]

S3 Fig

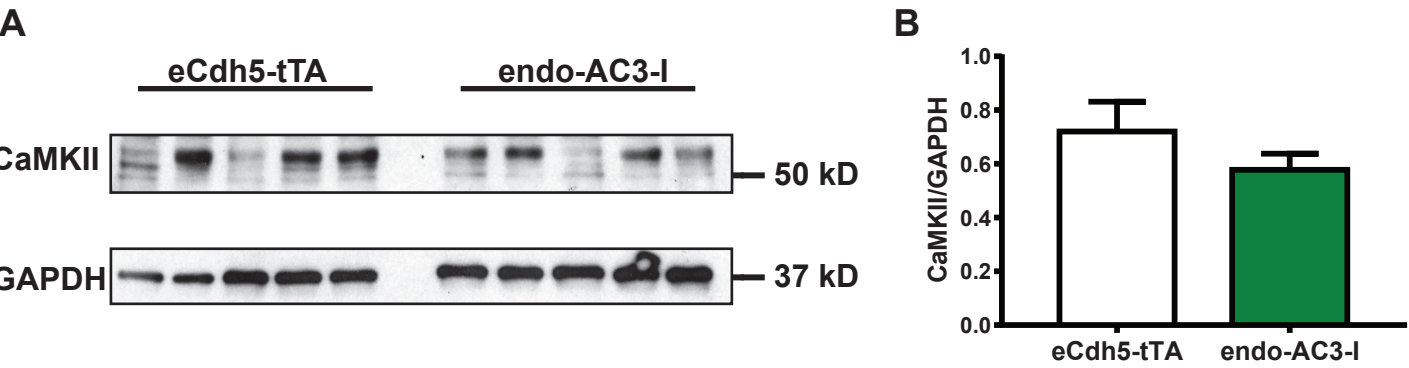

Supplement: S3 Fig — (A) Immunoblots for total CaMKII protein in aortas of eCdh5-tTA control and endo-AC3-I mice (lysates from one mouse per lane). (B) Quantiifcation of data in (A). Mean±SEM, n = 10 mice/group. (PDF) [file pone.0186311.s003.pdf]

S4 Fig

A

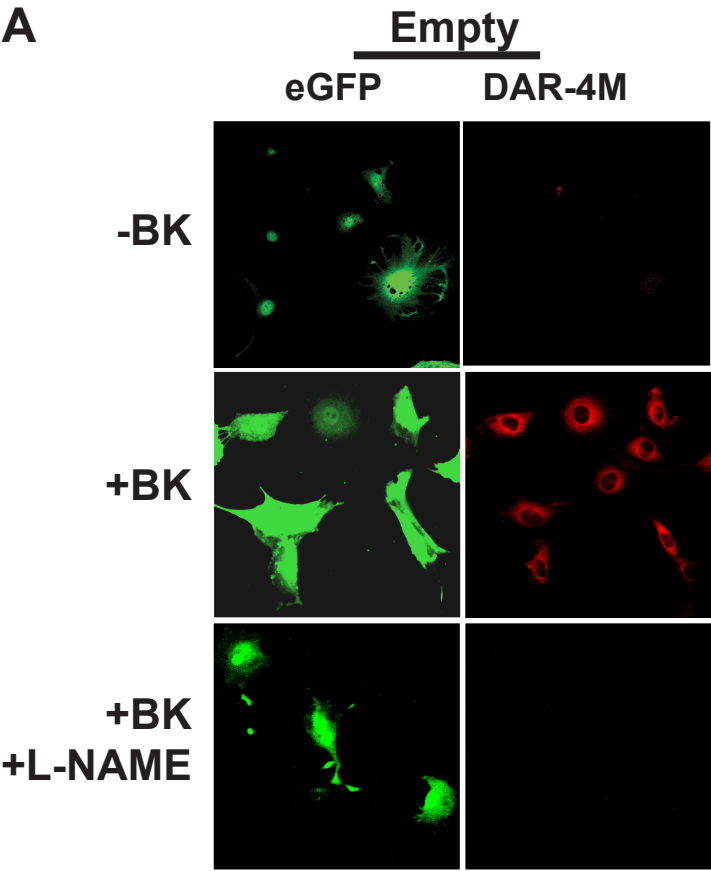

B

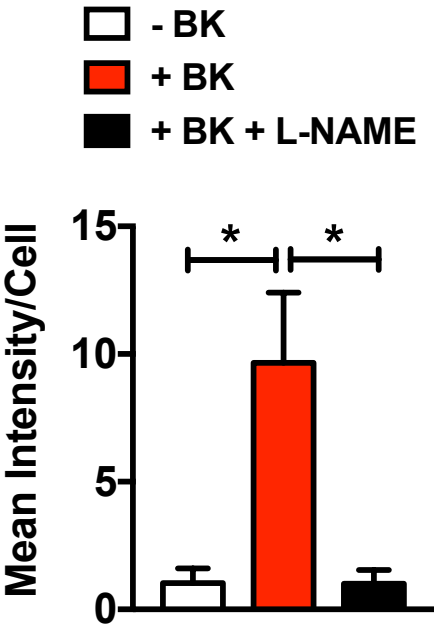

Supplement: S4 Fig — NO-sensitive DAR-4M FM fluorescence in bovine aortic endothelial cells infected with control adenovirus Ad5.CMV.IRES.eGFP.Empty (control adenovirus expresses eGFP). Treatment with 1μM bradykinin. Additional samples were pretreated with 100μM L-NAME for 30 minutes. Green: eGFP; red: DAR-4M FM. B. Densitometric analyses of the DAR-4M FM signal. Data are the average of 3 independent experiments. * p<0.05 vs.—BK. (PDF) [file pone.0186311.s004.pdf]
